# Foreign Correspondence

**Published:** 1889-03

**Authors:** 


### Foreign Correspondence.

To the EDITOR :

I had the honor to announce to the Dental Section of the International Medical Congress, at Washington, that the dentists of France deem the time of the holding of the "Exposition Universelle" a suitable occasion for the reunion of the dentists of all nations for the consideration of scientific and practical questions of interest to the profession.

In assembling this congress the French dentists do not wish to interfere in any way with the Dental Section of the International Medical Congress to be held in Berlin. In the domain of scientific and professional knowledge, emulation and progress are achieved by union and concourse of all. It seems to us, however, that there is a scientific and practical advantage to dentists in having a congress of their own. Our art is far reaching enough and of sufficient special importance for dentists to hold assemblies by and for themselves. We do not lose sight of the points that are of common interest to the general practitioner and the dentist, but in our eyes they are not sufficiently numerous to necessitate the union of the two in one general congress.

These reasons seem sufficient to justify the calling of the First International Dental Congress.

In order to facilitate the division of the work, and for the proper examination of the various communications, the committee of organization considers it best to divide the congress into sections. It has also been considered advantageous to concentrate the attention of the members of the congress upon questions determined upon in advance—leaving it to the congress to introduce others if thought best.

The following sections and questions have been arranged :

SECTION FIRST :—*Anatomy, Physiology, and Pathology.*

1. The teeth of different races.
2. The "rôle" of micro-organisms in dental and oral pathology.
3. The influence of nutrition in producing and resisting dental caries.
4. Dental and oral classification and terminology.

SECOND SECTION :—*Operative and Therapeutic Dentistry.*

1. Treatment of teeth with diseased and dead pulps.
2. Comparative value of gold and plastic materials for filling teeth, with observations on recent progress in this direction.
3. Local anæsthesia.

THIRD SECTION :—*Prosthetic Dentistry and Oral Deformities.*

1. Artificial crowns, bridge work, when indicated, and manner of construction.

2. Irregularities of the teeth and alveolar arches, with new methods of treatment.

3. Choice of material for artificial dentures.

4. Restoration of the face and jaws.

FOURTH SECTION :—*Dental Education and Hygiene.*

1. Instructions in dental art, methods, length of studies.

2d. Dental and oral hygiene during the process of dentition.

In order that the discussion may take the widest range and that the majority of the congress may be acquainted with the principal ideas of the communications to come before the congress, it has been decided to translate into French all papers and put them in print, provided they are received before July 1st, and do not exceed six printed pages in length. By so doing, all can come to the meetings having the text for the discussions.

It will be seen that the organizers of the International Dental Congress of 1889 have labored to the best of their ability to make this grand reunion profitable, not only to themselves, but to the great number of visiting dentists who will comprise no small part of the congress.

It was at first proposed to convene the congress on August 15th; but on learning that the meeting of the American Dental Association takes place at about that time and would prevent, most likely, many from attending, it has been decided to change the time to September 1st, in order to give our *American confreres* an opportunity to be present and co-operate with us.

It will be an excellent time to see Paris during the exposition. Living in France is cheap. The committee propose to furnish visiting dentists with a list of hotels agreeing to accommodate them at a price fixed in advance. It is thought houses can be found that will furnish excellent accommodations for three or four dollars a day.

The officers of the commission of organization are:

DR. DAVID, *President.*

DR. BRASSEUR, *Vice-President* (deceased).

DR. SAUSSINE, *Vice-President.*

DR. POURCHET, *General Secretary.*

DR. KUHN, *Treasurer.*

DR. BLOCKMAN, *Secretary.*

DR. DAMAIN, *Secretary.*

DR. GODON, *Secretary.*

All communications should be addressed to the General Secretary, M. Pourchet, 24 Rue de la Chauseé d'Antin. I shall also be happy to give your readers any information in my power. Address, Bureau de l'Odontologie, 2 Rue d'Amsterdam.

It is with great regret that I have to announce the sudden death of our most worthy associate, M. Brasseur, whom you had the good fortune to secure as correspondent for the INTERNATIONAL DENTAL JOURNAL. He was greatly esteemed by the French dentists. He was much interested in the use of hot air in therapeutic dentistry, and he had but recently devised a perfect electric injector.

In my next letter I shall speak more particularly of professional affairs in France.

(Signed) P. DUBOIS,

Feb. 4, 1889.

*President of the Odontological Society of Paris.*

Translated by DWIGHT M. CLAPP.

---

To the EDITOR:

I want to use some very grateful expression as my heading, but fail to find one which will convey the gratitude your friends over here feel for the splendid New Year's present you gave us in the Topical Index for the 1888 volume. As there seems to be a feeling that your journal, coming out fifteen days after the *Dental Cosmos* did with its monthly topical index, had perhaps taken the cue from that Journal, I may remind you that you wrote me on December 18th, 1888, that you had a complete topical cross index prepared for Volume IX (such as I had insisted upon the necessity of early in your administration), and that it would be added to the January No. You will, no doubt, receive the blessings of multitudes of new subscribers who have spent hours in fruitless search for some little thing which would be worth the price of the volume to them, if they could only find it.

What will your next surprise be?

I am glad to hear you intend to illustrate freely all articles which require it.

L. C. BRYAN.

Basel, February 4, 1889.

